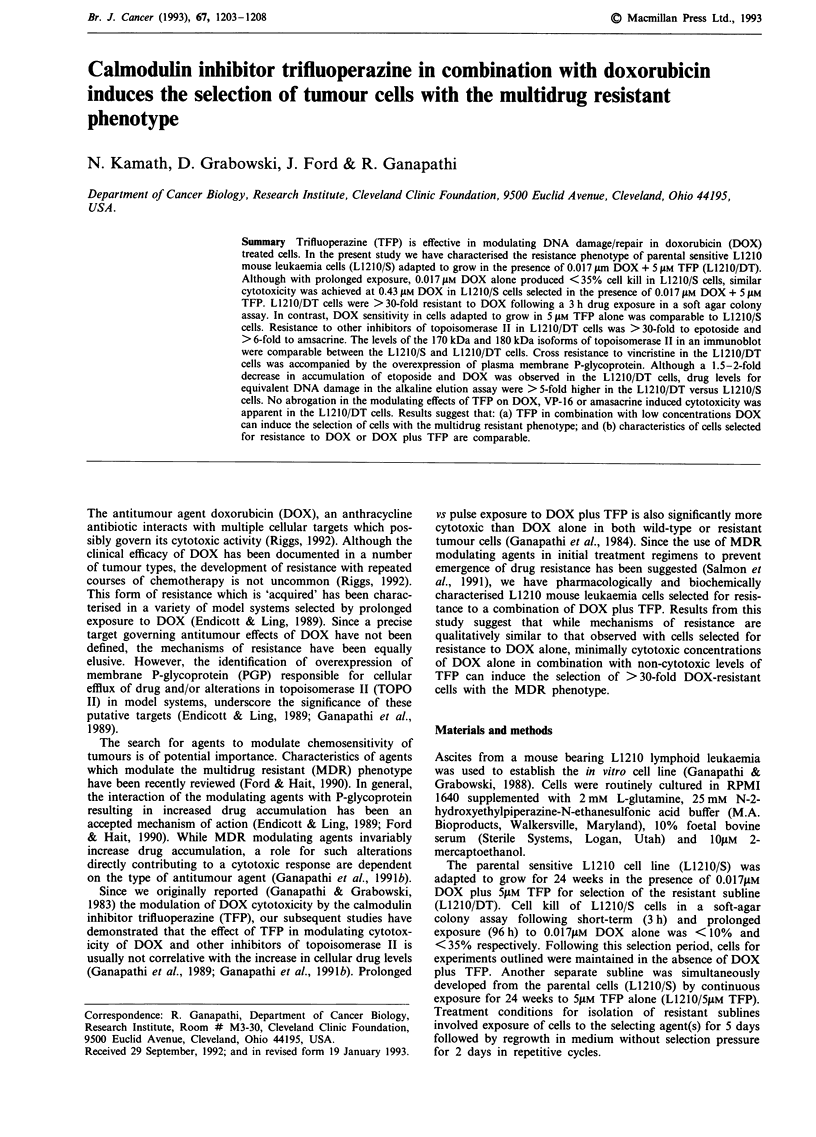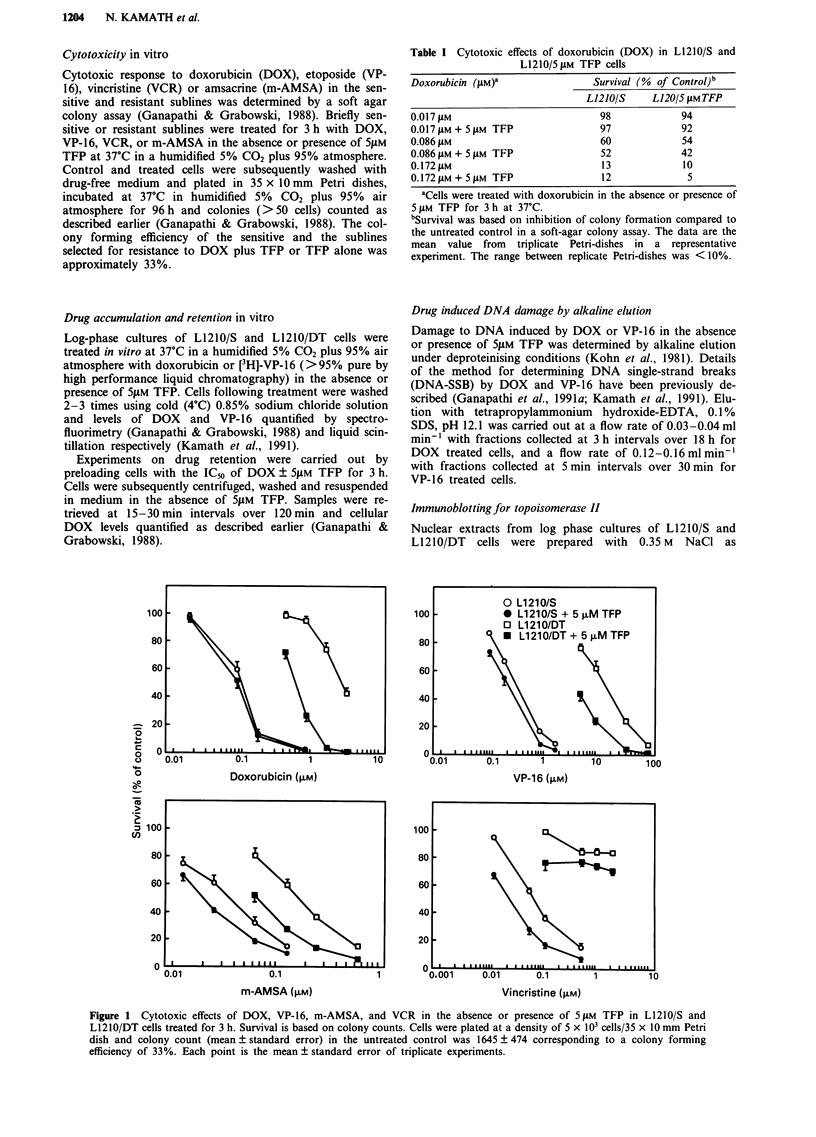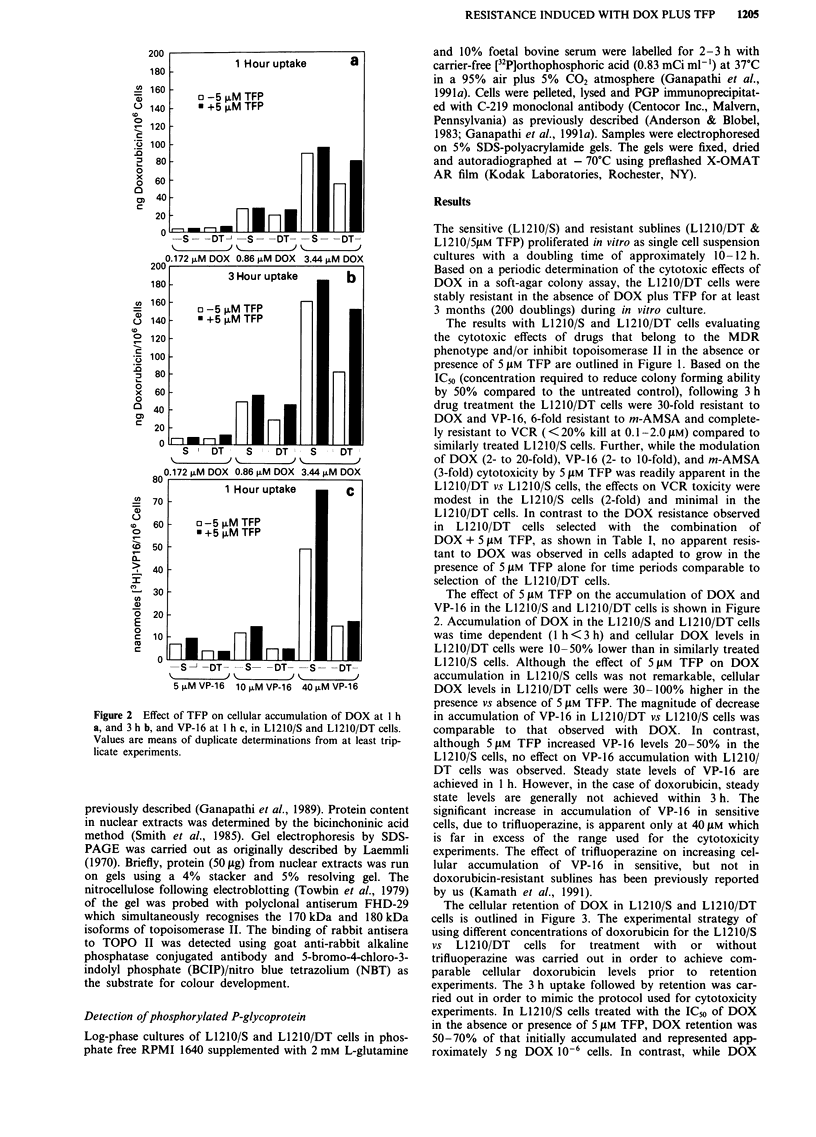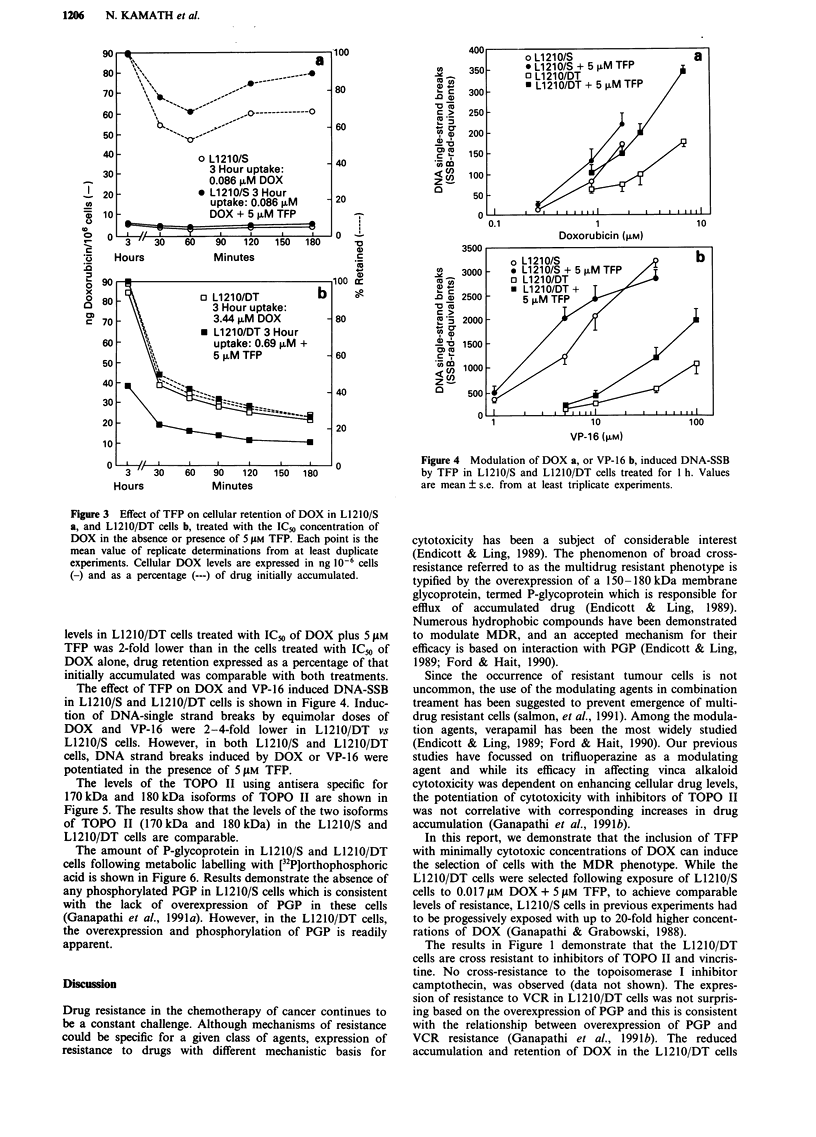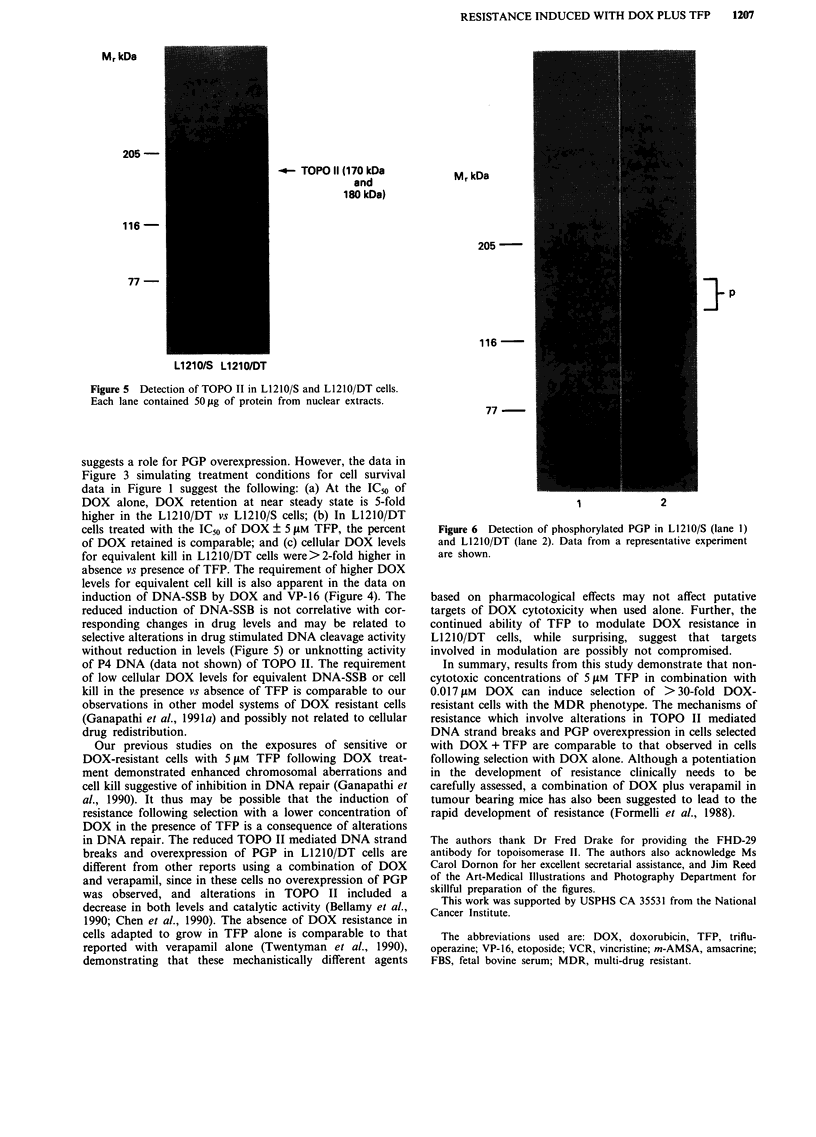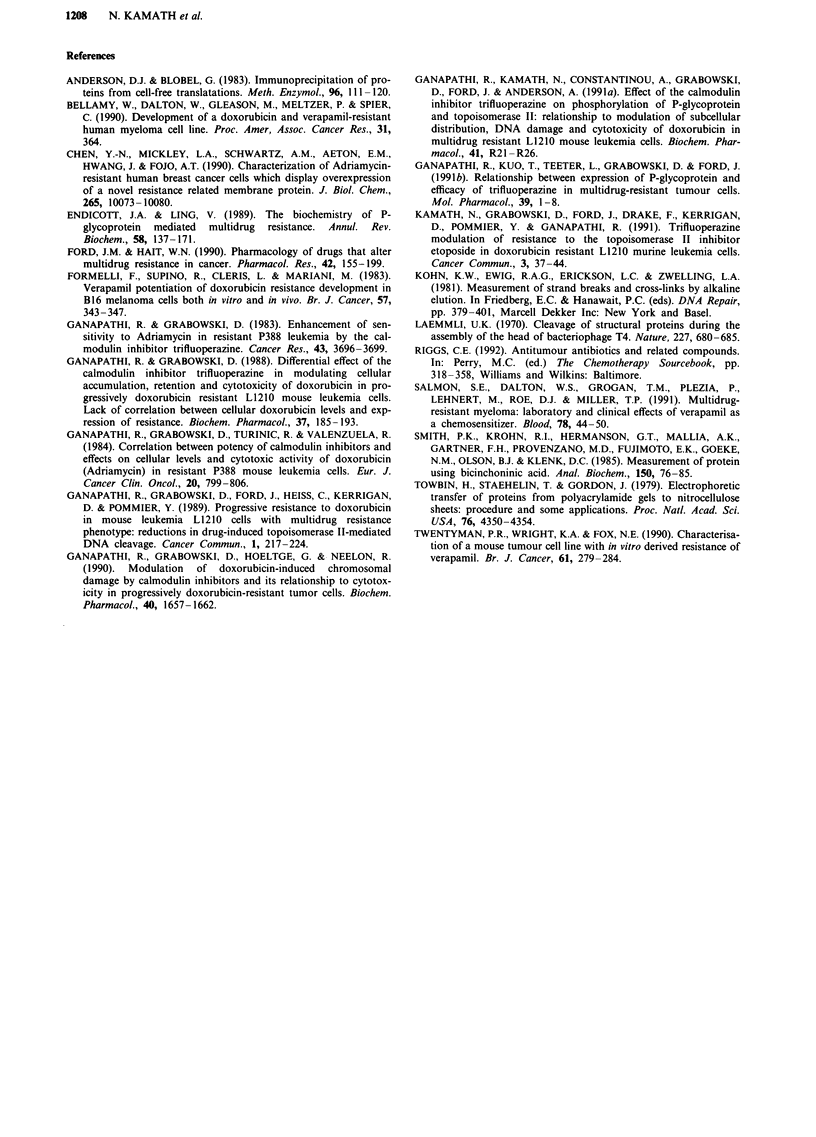# Calmodulin inhibitor trifluoperazine in combination with doxorubicin induces the selection of tumour cells with the multidrug resistant phenotype.

**DOI:** 10.1038/bjc.1993.226

**Published:** 1993-06

**Authors:** N. Kamath, D. Grabowski, J. Ford, R. Ganapathi

**Affiliations:** Department of Cancer Biology, Cleveland Clinic Foundation, Ohio 44195.

## Abstract

**Images:**


Br. J. Cancer (1993), 67, 1203-1208 © Macmillan Press Ltd., 1993

### Calmodulin inhibitor trifluoperazine in combination with doxorubicin induces the selection of tumour cells with the multidrug resistant phenotype

N. Kamath, D. Grabowski, J. Ford & R. Ganapathi

*Department of Cancer Biology, Research Institute, Cleveland Clinic Foundation, 9500 Euclid Avenue, Cleveland, Ohio 44195, USA.*

**Summary** Trifluoperazine (TFP) is effective in modulating DNA damage/repair in doxorubicin (DOX) treated cells. In the present study we have characterised the resistance phenotype of parental sensitive L1210 mouse leukaemia cells (L1210/S) adapted to grow in the presence of 0.017 μm DOX + 5 μM TFP (L1210/DT). Although with prolonged exposure, 0.017 μM DOX alone produced <35% cell kill in L1210/S cells, similar cytotoxicity was achieved at 0.43 μM DOX in L1210/S cells selected in the presence of 0.017 μM DOX + 5 μM TFP. L1210/DT cells were >30-fold resistant to DOX following a 3 h drug exposure in a soft agar colony assay. In contrast, DOX sensitivity in cells adapted to grow in 5 μM TFP alone was comparable to L1210/S cells. Resistance to other inhibitors of topoisomerase II in L1210/DT cells was >30-fold to epotoside and >6-fold to amsacrine. The levels of the 170 kDa and 180 kDa isoforms of topoisomerase II in an immunoblot were comparable between the L1210/S and L1210/DT cells. Cross resistance to vincristine in the L1210/DT cells was accompanied by the overexpression of plasma membrane P-glycoprotein. Although a 1.5-2-fold decrease in accumulation of etoposide and DOX was observed in the L1210/DT cells, drug levels for equivalent DNA damage in the alkaline elution assay were >5-fold higher in the L1210/DT versus L1210/S cells. No abrogation in the modulating effects of TFP on DOX, VP-16 or amasacrine induced cytotoxicity was apparent in the L1210/DT cells. Results suggest that: (a) TFP in combination with low concentrations DOX can induce the selection of cells with the multidrug resistant phenotype; and (b) characteristics of cells selected for resistance to DOX or DOX plus TFP are comparable.

The antitumour agent doxorubicin (DOX), an anthracycline antibiotic interacts with multiple cellular targets which possibly govern its cytotoxic activity (Riggs, 1992). Although the clinical efficacy of DOX has been documented in a number of tumour types, the development of resistance with repeated courses of chemotherapy is not uncommon (Riggs, 1992). This form of resistance which is 'acquired' has been characterised in a variety of model systems selected by prolonged exposure to DOX (Endicott & Ling, 1989). Since a precise target governing antitumour effects of DOX have not been defined, the mechanisms of resistance have been equally elusive. However, the identification of overexpression of membrane P-glycoprotein (PGP) responsible for cellular efflux of drug and/or alterations in topoisomerase II (TOPO II) in model systems, underscore the significance of these putative targets (Endicott & Ling, 1989; Ganapathi *et al.*, 1989).

The search for agents to modulate chemosensitivity of tumours is of potential importance. Characteristics of agents which modulate the multidrug resistant (MDR) phenotype have been recently reviewed (Ford & Hait, 1990). In general, the interaction of the modulating agents with P-glycoprotein resulting in increased drug accumulation has been an accepted mechanism of action (Endicott & Ling, 1989; Ford & Hait, 1990). While MDR modulating agents invariably increase drug accumulation, a role for such alterations directly contributing to a cytotoxic response are dependent on the type of antitumour agent (Ganapathi *et al.*, 1991*b*).

Since we originally reported (Ganapathi & Grabowski, 1983) the modulation of DOX cytotoxicity by the calmodulin inhibitor trifluoperazine (TFP), our subsequent studies have demonstrated that the effect of TFP in modulating cytotoxicity of DOX and other inhibitors of topoisomerase II is usually not correlative with the increase in cellular drug levels (Ganapathi *et al.*, 1989; Ganapathi *et al.*, 1991*b*). Prolonged *vs* pulse exposure to DOX plus TFP is also significantly more cytotoxic than DOX alone in both wild-type or resistant tumour cells (Ganapathi *et al.*, 1984). Since the use of MDR modulating agents in initial treatment regimens to prevent emergence of drug resistance has been suggested (Salmon *et al.*, 1991), we have pharmacologically and biochemically characterised L1210 mouse leukaemia cells selected for resistance to a combination of DOX plus TFP. Results from this study suggest that while mechanisms of resistance are qualitatively similar to that observed with cells selected for resistance to DOX alone, minimally cytotoxic concentrations of DOX alone in combination with non-cytotoxic levels of TFP can induce the selection of >30-fold DOX-resistant cells with the MDR phenotype.

### Materials and methods

Ascites from a mouse bearing L1210 lymphoid leukaemia was used to establish the *in vitro* cell line (Ganapathi & Grabowski, 1988). Cells were routinely cultured in RPMI 1640 supplemented with 2 mM L-glutamine, 25 mM N-2-hydroxyethylpiperazine-N-ethanesulfonic acid buffer (M.A. Bioproducts, Walkersville, Maryland), 10% foetal bovine serum (Sterile Systems, Logan, Utah) and 10μM 2-mercaptoethanol.

The parental sensitive L1210 cell line (L1210/S) was adapted to grow for 24 weeks in the presence of 0.017μM DOX plus 5μM TFP for selection of the resistant subline (L1210/DT). Cell kill of L1210/S cells in a soft-agar colony assay following short-term (3 h) and prolonged exposure (96 h) to 0.017μM DOX alone was <10% and <35% respectively. Following this selection period, cells for experiments outlined were maintained in the absence of DOX plus TFP. Another separate subline was simultaneously developed from the parental cells (L1210/S) by continuous exposure for 24 weeks to 5μM TFP alone (L1210/5μM TFP). Treatment conditions for isolation of resistant sublines involved exposure of cells to the selecting agent(s) for 5 days followed by regrowth in medium without selection pressure for 2 days in repetitive cycles.

Correspondence: R. Ganapathi, Department of Cancer Biology, Research Institute, Room # M3-30, Cleveland Clinic Foundation, 9500 Euclid Avenue, Cleveland, Ohio 44195, USA.
Received 29 September, 1992; and in revised form 19 January 1993.

*Cytotoxicity* in vitro

Cytotoxic response to doxorubicin (DOX), etoposide (VP-16), vincristine (VCR) or amsacrine (m-AMSA) in the sensitive and resistant sublines was determined by a soft agar colony assay (Ganapathi & Grabowski, 1988). Briefly sensitive or resistant sublines were treated for 3 h with DOX, VP-16, VCR, or m-AMSA in the absence or presence of 5μM TFP at 37°C in a humidified 5% $CO_2$ plus 95% atmosphere. Control and treated cells were subsequently washed with drug-free medium and plated in 35 × 10 mm Petri dishes, incubated at 37°C in humidified 5% $CO_2$ plus 95% air atmosphere for 96 h and colonies (>50 cells) counted as described earlier (Ganapathi & Grabowski, 1988). The colony forming efficiency of the sensitive and the sublines selected for resistance to DOX plus TFP or TFP alone was approximately 33%.

*Drug accumulation and retention* in vitro

Log-phase cultures of L1210/S and L1210/DT cells were treated *in vitro* at 37°C in a humidified 5% $CO_2$ plus 95% air atmosphere with doxorubicin or [$^3$H]-VP-16 (>95% pure by high performance liquid chromatography) in the absence or presence of 5μM TFP. Cells following treatment were washed 2–3 times using cold (4°C) 0.85% sodium chloride solution and levels of DOX and VP-16 quantified by spectrofluorimetry (Ganapathi & Grabowski, 1988) and liquid scintillation respectively (Kamath *et al.*, 1991).

Experiments on drug retention were carried out by preloading cells with the $IC_{50}$ of DOX ± 5μM TFP for 3 h. Cells were subsequently centrifuged, washed and resuspended in medium in the absence of 5μM TFP. Samples were retrieved at 15–30 min intervals over 120 min and cellular DOX levels quantified as described earlier (Ganapathi & Grabowski, 1988).

**Table I** Cytotoxic effects of doxorubicin (DOX) in L1210/S and L1210/5 μM TFP cells

| *Doxorubicin (μM)*[a] | *Survival (% of Control)*[b] | |
|---|---|---|
| | *L1210/S* | *L120/5 μMTFP* |
| 0.017 μM | 98 | 94 |
| 0.017 μM + 5 μM TFP | 97 | 92 |
| 0.086 μM | 60 | 54 |
| 0.086 μM + 5 μM TFP | 52 | 42 |
| 0.172 μM | 13 | 10 |
| 0.172 μM + 5 μM TFP | 12 | 5 |

[a]Cells were treated with doxorubicin in the absence or presence of 5 μM TFP for 3 h at 37°C.
[b]Survival was based on inhibition of colony formation compared to the untreated control in a soft-agar colony assay. The data are the mean value from triplicate Petri-dishes in a representative experiment. The range between replicate Petri-dishes was <10%.

*Drug induced DNA damage by alkaline elution*

Damage to DNA induced by DOX or VP-16 in the absence or presence of 5μM TFP was determined by alkaline elution under deproteinising conditions (Kohn *et al.*, 1981). Details of the method for determining DNA single-strand breaks (DNA-SSB) by DOX and VP-16 have been previously described (Ganapathi *et al.*, 1991*a*; Kamath *et al.*, 1991). Elution with tetrapropylammonium hydroxide-EDTA, 0.1% SDS, pH 12.1 was carried out at a flow rate of 0.03–0.04 ml $min^{-1}$ with fractions collected at 3 h intervals over 18 h for DOX treated cells, and a flow rate of 0.12–0.16 ml $min^{-1}$ with fractions collected at 5 min intervals over 30 min for VP-16 treated cells.

*Immunoblotting for topoisomerase II*

Nuclear extracts from log phase cultures of L1210/S and L1210/DT cells were prepared with 0.35 M NaCl as

**Figure 1** Cytotoxic effects of DOX, VP-16, m-AMSA, and VCR in the absence or presence of 5 μM TFP in L1210/S and L1210/DT cells treated for 3 h. Survival is based on colony counts. Cells were plated at a density of 5 × $10^3$ cells/35 × 10 mm Petri dish and colony count (mean ± standard error) in the untreated control was 1645 ± 474 corresponding to a colony forming efficiency of 33%. Each point is the mean ± standard error of triplicate experiments.

**Figure 2** Effect of TFP on cellular accumulation of DOX at 1 h **a**, and 3 h **b**, and VP-16 at 1 h **c**, in L1210/S and L1210/DT cells. Values are means of duplicate determinations from at least triplicate experiments.

previously described (Ganapathi *et al.*, 1989). Protein content in nuclear extracts was determined by the bicinchoninic acid method (Smith *et al.*, 1985). Gel electrophoresis by SDS-PAGE was carried out as originally described by Laemmli (1970). Briefly, protein (50 μg) from nuclear extracts was run on gels using a 4% stacker and 5% resolving gel. The nitrocellulose following electroblotting (Towbin *et al.*, 1979) of the gel was probed with polyclonal antiserum FHD-29 which simultaneously recognises the 170 kDa and 180 kDa isoforms of topoisomerase II. The binding of rabbit antisera to TOPO II was detected using goat anti-rabbit alkaline phosphatase conjugated antibody and 5-bromo-4-chloro-3-indolyl phosphate (BCIP)/nitro blue tetrazolium (NBT) as the substrate for colour development.

### *Detection of phosphorylated P-glycoprotein*

Log-phase cultures of L1210/S and L1210/DT cells in phosphate free RPMI 1640 supplemented with 2 mM L-glutamine and 10% foetal bovine serum were labelled for 2–3 h with carrier-free [$^{32}$P]orthophosphoric acid (0.83 mCi ml$^{-1}$) at 37°C in a 95% air plus 5% $CO_2$ atmosphere (Ganapathi *et al.*, 1991*a*). Cells were pelleted, lysed and PGP immunoprecipitated with C-219 monoclonal antibody (Centocor Inc., Malvern, Pennsylvania) as previously described (Anderson & Blobel, 1983; Ganapathi *et al.*, 1991*a*). Samples were electrophoresed on 5% SDS-polyacrylamide gels. The gels were fixed, dried and autoradiographed at − 70°C using preflashed X-OMAT AR film (Kodak Laboratories, Rochester, NY).

### Results

The sensitive (L1210/S) and resistant sublines (L1210/DT & L1210/5μM TFP) proliferated *in vitro* as single cell suspension cultures with a doubling time of approximately 10–12 h. Based on a periodic determination of the cytotoxic effects of DOX in a soft-agar colony assay, the L1210/DT cells were stably resistant in the absence of DOX plus TFP for at least 3 months (200 doublings) during *in vitro* culture.

The results with L1210/S and L1210/DT cells evaluating the cytotoxic effects of drugs that belong to the MDR phenotype and/or inhibit topoisomerase II in the absence or presence of 5 μM TFP are outlined in Figure 1. Based on the $IC_{50}$ (concentration required to reduce colony forming ability by 50% compared to the untreated control), following 3 h drug treatment the L1210/DT cells were 30-fold resistant to DOX and VP-16, 6-fold resistant to *m*-AMSA and completely resistant to VCR (< 20% kill at 0.1–2.0 μM) compared to similarly treated L1210/S cells. Further, while the modulation of DOX (2- to 20-fold), VP-16 (2- to 10-fold), and *m*-AMSA (3-fold) cytotoxicity by 5 μM TFP was readily apparent in the L1210/DT *vs* L1210/S cells, the effects on VCR toxicity were modest in the L1210/S cells (2-fold) and minimal in the L1210/DT cells. In contrast to the DOX resistance observed in L1210/DT cells selected with the combination of DOX + 5 μM TFP, as shown in Table I, no apparent resistant to DOX was observed in cells adapted to grow in the presence of 5 μM TFP alone for time periods comparable to selection of the L1210/DT cells.

The effect of 5 μM TFP on the accumulation of DOX and VP-16 in the L1210/S and L1210/DT cells is shown in Figure 2. Accumulation of DOX in the L1210/S and L1210/DT cells was time dependent (1 h < 3 h) and cellular DOX levels in L1210/DT cells were 10–50% lower than in similarly treated L1210/S cells. Although the effect of 5 μM TFP on DOX accumulation in L1210/S cells was not remarkable, cellular DOX levels in L1210/DT cells were 30–100% higher in the presence *vs* absence of 5 μM TFP. The magnitude of decrease in accumulation of VP-16 in L1210/DT *vs* L1210/S cells was comparable to that observed with DOX. In contrast, although 5 μM TFP increased VP-16 levels 20–50% in the L1210/S cells, no effect on VP-16 accumulation with L1210/DT cells was observed. Steady state levels of VP-16 are achieved in 1 h. However, in the case of doxorubicin, steady state levels are generally not achieved within 3 h. The significant increase in accumulation of VP-16 in sensitive cells, due to trifluoperazine, is apparent only at 40 μM which is far in excess of the range used for the cytotoxicity experiments. The effect of trifluoperazine on increasing cellular accumulation of VP-16 in sensitive, but not in doxorubicin-resistant sublines has been previously reported by us (Kamath *et al.*, 1991).

The cellular retention of DOX in L1210/S and L1210/DT cells is outlined in Figure 3. The experimental strategy of using different concentrations of doxorubicin for the L1210/S *vs* L1210/DT cells for treatment with or without trifluoperazine was carried out in order to achieve comparable cellular doxorubicin levels prior to retention experiments. The 3 h uptake followed by retention was carried out in order to mimic the protocol used for cytotoxicity experiments. In L1210/S cells treated with the $IC_{50}$ of DOX in the absence or presence of 5 μM TFP, DOX retention was 50–70% of that initially accumulated and represented approximately 5 ng DOX $10^{-6}$ cells. In contrast, while DOX

Figure 3 Effect of TFP on cellular retention of DOX in L1210/S **a**, and L1210/DT cells **b**, treated with the $IC_{50}$ concentration of DOX in the absence or presence of 5 µM TFP. Each point is the mean value of replicate determinations from at least duplicate experiments. Cellular DOX levels are expressed in ng $10^{-6}$ cells (–) and as a percentage (---) of drug initially accumulated.

Figure 4 Modulation of DOX **a**, or VP-16 **b**, induced DNA-SSB by TFP in L1210/S and L1210/DT cells treated for 1 h. Values are mean ± s.e. from at least triplicate experiments.

levels in L1210/DT cells treated with $IC_{50}$ of DOX plus 5 µM TFP was 2-fold lower than in the cells treated with $IC_{50}$ of DOX alone, drug retention expressed as a percentage of that initially accumulated was comparable with both treatments.

The effect of TFP on DOX and VP-16 induced DNA-SSB in L1210/S and L1210/DT cells is shown in Figure 4. Induction of DNA-single strand breaks by equimolar doses of DOX and VP-16 were 2–4-fold lower in L1210/DT *vs* L1210/S cells. However, in both L1210/S and L1210/DT cells, DNA strand breaks induced by DOX or VP-16 were potentiated in the presence of 5 µM TFP.

The levels of the TOPO II using antisera specific for 170 kDa and 180 kDa isoforms of TOPO II are shown in Figure 5. The results show that the levels of the two isoforms of TOPO II (170 kDa and 180 kDa) in the L1210/S and L1210/DT cells are comparable.

The amount of P-glycoprotein in L1210/S and L1210/DT cells following metabolic labelling with [$^{32}$P]orthophosphoric acid is shown in Figure 6. Results demonstrate the absence of any phosphorylated PGP in L1210/S cells which is consistent with the lack of overexpression of PGP in these cells (Ganapathi *et al.*, 1991*a*). However, in the L1210/DT cells, the overexpression and phosphorylation of PGP is readily apparent.

### Discussion

Drug resistance in the chemotherapy of cancer continues to be a constant challenge. Although mechanisms of resistance could be specific for a given class of agents, expression of resistance to drugs with different mechanistic basis for cytotoxicity has been a subject of considerable interest (Endicott & Ling, 1989). The phenomenon of broad cross-resistance referred to as the multidrug resistant phenotype is typified by the overexpression of a 150–180 kDa membrane glycoprotein, termed P-glycoprotein which is responsible for efflux of accumulated drug (Endicott & Ling, 1989). Numerous hydrophobic compounds have been demonstrated to modulate MDR, and an accepted mechanism for their efficacy is based on interaction with PGP (Endicott & Ling, 1989; Ford & Hait, 1990).

Since the occurrence of resistant tumour cells is not uncommon, the use of the modulating agents in combination treament has been suggested to prevent emergence of multidrug resistant cells (salmon, *et al.*, 1991). Among the modulation agents, verapamil has been the most widely studied (Endicott & Ling, 1989; Ford & Hait, 1990). Our previous studies have focussed on trifluoperazine as a modulating agent and while its efficacy in affecting vinca alkaloid cytotoxicity was dependent on enhancing cellular drug levels, the potentiation of cytotoxicity with inhibitors of TOPO II was not correlative with corresponding increases in drug accumulation (Ganapathi *et al.*, 1991*b*).

In this report, we demonstrate that the inclusion of TFP with minimally cytotoxic concentrations of DOX can induce the selection of cells with the MDR phenotype. While the L1210/DT cells were selected following exposure of L1210/S cells to 0.017 µM DOX + 5 µM TFP, to achieve comparable levels of resistance, L1210/S cells in previous experiments had to be progessively exposed with up to 20-fold higher concentrations of DOX (Ganapathi & Grabowski, 1988).

The results in Figure 1 demonstrate that the L1210/DT cells are cross resistant to inhibitors of TOPO II and vincristine. No cross-resistance to the topoisomerase I inhibitor camptothecin, was observed (data not shown). The expression of resistance to VCR in L1210/DT cells was not surprising based on the overexpression of PGP and this is consistent with the relationship between overexpression of PGP and VCR resistance (Ganapathi *et al.*, 1991*b*). The reduced accumulation and retention of DOX in the L1210/DT cells

**Figure 5** Detection of TOPO II in L1210/S and L1210/DT cells. Each lane contained 50 $\mu$g of protein from nuclear extracts.

**Figure 6** Detection of phosphorylated PGP in L1210/S (lane 1) and L1210/DT (lane 2). Data from a representative experiment are shown.

suggests a role for PGP overexpression. However, the data in Figure 3 simulating treatment conditions for cell survival data in Figure 1 suggest the following: (a) At the $IC_{50}$ of DOX alone, DOX retention at near steady state is 5-fold higher in the L1210/DT *vs* L1210/S cells; (b) In L1210/DT cells treated with the $IC_{50}$ of DOX $\pm$ 5 $\mu$M TFP, the percent of DOX retained is comparable; and (c) cellular DOX levels for equivalent kill in L1210/DT cells were >2-fold higher in absence *vs* presence of TFP. The requirement of higher DOX levels for equivalent cell kill is also apparent in the data on induction of DNA-SSB by DOX and VP-16 (Figure 4). The reduced induction of DNA-SSB is not correlative with corresponding changes in drug levels and may be related to selective alterations in drug stimulated DNA cleavage activity without reduction in levels (Figure 5) or unknotting activity of P4 DNA (data not shown) of TOPO II. The requirement of low cellular DOX levels for equivalent DNA-SSB or cell kill in the presence *vs* absence of TFP is comparable to our observations in other model systems of DOX resistant cells (Ganapathi *et al.*, 1991*a*) and possibly not related to cellular drug redistribution.

Our previous studies on the exposures of sensitive or DOX-resistant cells with 5 $\mu$M TFP following DOX treatment demonstrated enhanced chromosomal aberrations and cell kill suggestive of inhibition in DNA repair (Ganapathi *et al.*, 1990). It thus may be possible that the induction of resistance following selection with a lower concentration of DOX in the presence of TFP is a consequence of alterations in DNA repair. The reduced TOPO II mediated DNA strand breaks and overexpression of PGP in L1210/DT cells are different from other reports using a combination of DOX and verapamil, since in these cells no overexpression of PGP was observed, and alterations in TOPO II included a decrease in both levels and catalytic activity (Bellamy *et al.*, 1990; Chen *et al.*, 1990). The absence of DOX resistance in cells adapted to grow in TFP alone is comparable to that reported with verapamil alone (Twentyman *et al.*, 1990), demonstrating that these mechanistically different agents based on pharmacological effects may not affect putative targets of DOX cytotoxicity when used alone. Further, the continued ability of TFP to modulate DOX resistance in L1210/DT cells, while surprising, suggest that targets involved in modulation are possibly not compromised.

In summary, results from this study demonstrate that non-cytotoxic concentrations of 5 $\mu$M TFP in combination with 0.017 $\mu$M DOX can induce selection of >30-fold DOX-resistant cells with the MDR phenotype. The mechanisms of resistance which involve alterations in TOPO II mediated DNA strand breaks and PGP overexpression in cells selected with DOX + TFP are comparable to that observed in cells following selection with DOX alone. Although a potentiation in the development of resistance clinically needs to be carefully assessed, a combination of DOX plus verapamil in tumour bearing mice has also been suggested to lead to the rapid development of resistance (Formelli *et al.*, 1988).

The authors thank Dr Fred Drake for providing the FHD-29 antibody for topoisomerase II. The authors also acknowledge Ms Carol Dornon for her excellent secretarial assistance, and Jim Reed of the Art-Medical Illustrations and Photography Department for skillful preparation of the figures.

This work was supported by USPHS CA 35531 from the National Cancer Institute.

The abbreviations used are: DOX, doxorubicin, TFP, trifluoperazine; VP-16, etoposide; VCR, vincristine; *m*-AMSA, amsacrine; FBS, fetal bovine serum; MDR, multi-drug resistant.

### References

ANDERSON, D.J. & BLOBEL, G. (1983). Immunoprecipitation of proteins from cell-free translatations. *Meth. Enzymol.*, **96**, 111–120.

BELLAMY, W., DALTON, W., GLEASON, M., MELTZER, P. & SPIER, C. (1990). Development of a doxorubicin and verapamil-resistant human myeloma cell line. *Proc. Amer, Assoc. Cancer Res.*, **31**, 364.

CHEN, Y.-N., MICKLEY, L.A., SCHWARTZ, A.M., AETON, E.M., HWANG, J. & FOJO, A.T. (1990). Characterization of Adriamycin-resistant human breast cancer cells which display overexpression of a novel resistance related membrane protein. *J. Biol. Chem.*, **265**, 10073–10080.

ENDICOTT, J.A. & LING, V. (1989). The biochemistry of P-glycoprotein mediated multidrug resistance. *Annul. Rev. Biochem.*, **58**, 137–171.

FORD, J.M. & HAIT, W.N. (1990). Pharmacology of drugs that alter multidrug resistance in cancer. *Pharmacol. Res.*, **42**, 155–199.

FORMELLI, F., SUPINO, R., CLERIS, L. & MARIANI, M. (1983). Verapamil potentiation of doxorubicin resistance development in B16 melanoma cells both *in vitro* and *in vivo*. *Br. J. Cancer*, **57**, 343–347.

GANAPATHI, R. & GRABOWSKI, D. (1983). Enhancement of sensitivity to Adriamycin in resistant P388 leukemia by the calmodulin inhibitor trifluoperazine. *Cancer Res.*, **43**, 3696–3699.

GANAPATHI, R. & GRABOWSKI, D. (1988). Differential effect of the calmodulin inhibitor trifluoperazine in modulating cellular accumulation, retention and cytotoxicity of doxorubicin in progressively doxorubicin resistant L1210 mouse leukemia cells. Lack of correlation between cellular doxorubicin levels and expression of resistance. *Biochem. Pharmacol.*, **37**, 185–193.

GANAPATHI, R., GRABOWSKI, D., TURINIC, R. & VALENZUELA, R. (1984). Correlation between potency of calmodulin inhibitors and effects on cellular levels and cytotoxic activity of doxorubicin (Adriamycin) in resistant P388 mouse leukemia cells. *Eur. J. Cancer Clin. Oncol.*, **20**, 799–806.

GANAPATHI, R., GRABOWSKI, D., FORD, J., HEISS, C., KERRIGAN, D. & POMMIER, Y. (1989). Progressive resistance to doxorubicin in mouse leukemia L1210 cells with multidrug resistance phenotype: reductions in drug-induced topoisomerase II-mediated DNA cleavage. *Cancer Commun.*, **1**, 217–224.

GANAPATHI, R., GRABOWSKI, D., HOELTGE, G. & NEELON, R. (1990). Modulation of doxorubicin-induced chromosomal damage by calmodulin inhibitors and its relationship to cytotoxicity in progressively doxorubicin-resistant tumor cells. *Biochem. Pharmacol.*, **40**, 1657–1662.

GANAPATHI, R., KAMATH, N., CONSTANTINOU, A., GRABOWSKI, D., FORD, J. & ANDERSON, A. (1991*a*). Effect of the calmodulin inhibitor trifluoperazine on phosphorylation of P-glycoprotein and topoisomerase II: relationship to modulation of subcellular distribution, DNA damage and cytotoxicity of doxorubicin in multidrug resistant L1210 mouse leukemia cells. *Biochem. Pharmacol.*, **41**, R21–R26.

GANAPATHI, R., KUO, T., TEETER, L., GRABOWSKI, D. & FORD, J. (1991*b*). Relationship between expression of P-glycoprotein and efficacy of trifluoperazine in multidrug-resistant tumour cells. *Mol. Pharmacol.*, **39**, 1–8.

KAMATH, N., GRABOWSKI, D., FORD, J., DRAKE, F., KERRIGAN, D., POMMIER, Y. & GANAPATHI, R. (1991). Trifluoperazine modulation of resistance to the topoisomerase II inhibitor etoposide in doxorubicin resistant L1210 murine leukemia cells. *Cancer Commun.*, **3**, 37–44.

KOHN, K.W., EWIG, R.A.G., ERICKSON, L.C. & ZWELLING, L.A. (1981). Measurement of strand breaks and cross-links by alkaline elution. In Friedberg, E.C. & Hanawait, P.C. (eds). *DNA Repair*, pp. 379–401, Marcell Dekker Inc: New York and Basel.

LAEMMLI, U.K. (1970). Cleavage of structural proteins during the assembly of the head of bacteriophage T4. *Nature*, 227, 680–685.

RIGGS, C.E. (1992). Antitumour antibiotics and related compounds. In: Perry, M.C. (ed.) *The Chemotherapy Sourcebook*, pp. 318–358, Williams and Wilkins: Baltimore.

SALMON, S.E., DALTON, W.S., GROGAN, T.M., PLEZIA, P., LEHNERT, M., ROE, D.J. & MILLER, T.P. (1991). Multidrug-resistant myeloma: laboratory and clinical effects of verapamil as a chemosensitizer. *Blood*, **78**, 44–50.

SMITH, P.K., KROHN, R.I., HERMANSON, G.T., MALLIA, A.K., GARTNER, F.H., PROVENZANO, M.D., FUJIMOTO, E.K., GOEKE, N.M., OLSON, B.J. & KLENK, D.C. (1985). Measurement of protein using bicinchoninic acid. *Anal. Biochem.*, **150**, 76–85.

TOWBIN, H., STAEHELIN, T. & GORDON, J. (1979). Electrophoretic transfer of proteins from polyacrylamide gels to nitrocellulose sheets: procedure and some applications. *Proc. Natl. Acad. Sci. USA*, **76**, 4350–4354.

TWENTYMAN, P.R., WRIGHT, K.A. & FOX, N.E. (1990). Characterisation of a mouse tumour cell line with *in vitro* derived resistance of verapamil. *Br. J. Cancer*, **61**, 279–284.